# Bovine leukemia virus transmission rates in persistent lymphocytotic infected dairy cows

**DOI:** 10.3389/fvets.2024.1367810

**Published:** 2024-07-17

**Authors:** Bibiana Benavides, Gustavo Monti

**Affiliations:** ^1^Animal Health Department, University of Nariño, San Juan de Pasto, Colombia; ^2^Quantitative Veterinary Epidemiology Group, Wageningen University, Wageningen, Netherlands

**Keywords:** dairy cattle, bovine leukemia virus, transmission, basic reproduction ratio, infectiousness, risk management

## Abstract

Bovine leukemia virus (BLV) establishes a lifelong persistent infection in dairy cattle. White blood cell count (WBC) is correlated with proviral load in the blood and milk of BLV-infected cattle, and testing WBC can be used to assess both BLV infectiousness levels and risk of BLV transmission from different types of infected animals. The objective of the study was to compare effective transmission rates (β) and the basic reproduction ratio (*R*_o_) among two types of BLV-infected dairy cows in Chile: those affected with persistent lymphocytosis (PL) vs. aleukemic (AL).The estimated (β) coefficient was higher in PL cattle [1.1; 95% Confidence interval (CI) (−1.6, 3.8)], compared to AL cattle (−3.1; 95% CI = −3.7, −2.5). In addition, the *R*_o_ was higher in PL cattle (60.4; 95% CI = 3.5; 820.6), compared to AL cattle (1.5; 95% CI = 0.7, 3.1). The ratio between PL/AL expected rate of cases was 73.9. The estimated effective transmission rate and the R_o_ were higher in PL cattle compared to AL cattle. The WBC test is a convenient alternative that can be considered for risk identification and risk management of BLV infection in dairy herds; particularly in livestock regions where laboratory capacity is limited (e.g., use of PCR or gene sequencing techniques) and/or molecular tests are not cost-effective. Therefore, when prevalence of infection is high, the removal of PL cattle should be engaged to control BLV within-herds.

## 1 Introduction

Bovine leukemia virus (BLV) is a retrovirus of the genus Deltaretrovirus, which causes lymphocytosis and fatal lymphosarcoma in bovine lymphoid tissues (1).

Bovine leukemia virus is transmitted through provirus-infected lymphocytes (2) via various routes. However, horizontal transmission is the most common route of infection, and occurs through the iatrogenic transfer of blood from infected cows to susceptible animals. These transfers arise because of herd management practices (3–5) or by hematophagous insects (6).

Cattle infected with BLV become lifelong virus carriers. Qualitative reporting using molecular techniques has highlighted the potential for differentials in infectiousness levels based on high or low proviral loads (7, 8).

Testing and culling approaches have been used in eradication programs worldwide. Frequent testing of all susceptible cattle and the culling of seropositive individuals are measures taken to reduce the prevalence of BLV and eventually eradicate the virus in infected herds (9). However, culling test-positive animals is often not economically feasible or practical in herds where BLV prevalence is high (10). In those situations, it has been proposed to focus on the strategic culling of high viral load cattle—as this may reduce transmission risk and lower within-herd prevalence to a level, at which it is possible to eliminate all test-positive animals (10).

For many industries worldwide, laboratory capacity is limited (e.g., use of PCR or gene sequencing) and/or these tests are not cost-effective for routine use in mass testing. Therefore, a simpler test is required that can be used as a proxy for risk identification and risk management of BLV infection in cattle—and the white blood cell count (WBC) test is a simple and known probe which can fulfill that purpose. Although few infected animals can have both a high proviral load and a normal lymphocyte count (8), cattle with persistent lymphocytosis (PL) are more likely to have a higher proviral load than cattle with a normal WBC (8, 11–14).

In addition, it is important to measure the impact of different infectiousness levels on transmission dynamics in animal populations, to assess the potential influence of such a control strategy. As information on varying degrees of infection among BLV-infected animals is unavailable, one approach is to obtain a quantitative measurement of the differential infectivity (15). Transmission can be measured in longitudinal field studies and expressed using the basic reproduction ratio (*R*_0_) metric, which is an essential parameter in the management of infectious diseases. *R*_0_ describes the average number of secondary cases within a susceptible population that one typical infectious individual will cause over the course of their infectious period (16). *R*_0_ has a threshold value of 1, and if *R*_0_ > 1, then minor and major outbreaks can occur, and an endemic situation may be established and maintained. However, when *R*_0_ < 1, an infection does not spread, and the infection will fade out (16, 17). For BLV, an overall *R*_0_ has been estimated (18)—but this was based on the assumption that all BLV infected animals are equally infectious.

As such, this study aimed to compare effective transmission rates (β) and the *R*_0_ among BLV-infected dairy cows in Chile affected by either PL or AL.

## 2 Materials and methods

### 2.1 Study herds

The study, conducted between 2006 and 2007, involved 1,527 dairy cows on 11 commercial dairy herds in Chile (1). Farms were selected based on background information obtained from a previous BLV study in Southern Chile that had sampled infected farms (19). Dairy cows included in this study were on pasture throughout the year, the main breed was Holstein and the group management practices that were considered were if bull-mounted, artificial insemination, rectal palpation, and use of injections with single or common needle for treatments or vaccination.

### 2.2 Study design

The study was designed as an observational cohort study with a follow-up period of 6 months, given that selected herds in this study tested and culled positive animals and an extended period would have resulted in a large follow-up loss and an inconvenience in reaching the required sample size. To estimate the sample size needed, we used the formula below (20), which considered the number of individuals (*N*) in each group by a difference in *p*_1_-*p*_2_ ratios between two groups over time:


$$N=\frac{{\left[Z_{\alpha }{\left(2\;pq\right)}^{1/2}+Z_{\beta }{\left(p_{1}q_{1}+p_{2}q_{2}\right)}^{1/2}\right]}^{2}\left(1+\left(n-1\right)\rho \right)}{n\;{\left(p_{1}-p_{2}\right)}^{2}}$$

Where:

*Z*_α_ = *Z* value for the error type I; *Z*_β_ = *Z* value for error type II; *p*_1_ = response ratio in exposed group (*q*_1_ = 1 − *p*_1_); *p*_2_ = response ratio in unexposed group (*q*_2_ = 1 − *p*_2_); *p* = (*p*_1_ + *p*_2_)/2; *q* = 1 – *p*; ρ is the expected common correlation between *n* observations.

Group 1 included infected animals that present with PL (*p*_1_), while group 2 (*p*_2_) were AL infected animals. We expected a proportion of infected animals for *p*_1_ = 35% and *p*_2_ = 5%, based on the potential of transmission of individuals according to their lymphocyte state (8). The correlation between samples was set at 0.6, and we assumed an error type I and II of 5 and 20%, respectively. Therefore, a minimum of 18 observations were required for each group. The study included 45 animals that developed PL, which exceeded the calculated sample size—and this ensured we would find (if it existed) the difference between the groups regarding transmission capacity according to their lymphocyte status.

Each cow was sampled monthly and tested for BLV antibodies, while each cow’s WBC was tested on day 0 and day 180.

### 2.3 Collection of blood samples

Blood samples (6 mL) were collected from the medium coccygeal vein via venipuncture, 3 mL was collected in EDTA tubes to perform the WBC and smears, and 3 mL collected in a tube without EDTA to obtain the serum of each animal, then refrigerated until they arrived at a designated laboratory at the Universidad Austral de Chile. Blood samples were centrifuged, and the serum samples were identified with a unique numeric code and stored in Eppendorf tubes at −20C until further processing (1).

### 2.4 Collection of milk samples

Composite milk samples (5 mL) of the four quarters were obtained from each study cow by farm personnel during milking. Milk samples were collected in test tubes and given a unique number, then refrigerated until they arrived at a designated laboratory at the Universidad Austral de Chile (1).

### 2.5 Laboratory procedures

Serum and milk samples were tested using commercial blocking ELISA kits (INGEZIM BLV COMPAC 2.0) based on the gp51 monoclonal antibodies, following the manufacturers’ recommended protocols. The test allows for the detection of specific antibodies in bovine serum or milk (1).

To perform the complete blood count, we used a Sysmex KX-21 N counter. We diluted blood samples per the ratio of four units of distilled water to one unit of blood, and the counter automatically provided the WBC/mm^3^. Smears were mounted on slides stained with Corzap quick dye, then viewed under a conventional microscope at a magnification of 100X to obtain the differential count. We counted 100 cells per smear and calculated the absolute value by multiplying the relative number of lymphocytes in the smear by the total number of leukocytes. The absolute lymphocyte count should be interpreted jointly with the animal’s age (21). Table 1 summarizes the European Community (EC) Key (Bendixen key) that was used for that purpose (22). A PL animal was defined as one that was infected and showed lymphocytosis in two blood samples taken 6 months apart. An AL animal was defined as one that was infected but either did not show lymphocytosis or only showed lymphocytosis in one blood sample.

**Table 1:** Key used for the detection of PL animals (based on the EC key).

Age (years)	Lymphocyte counts (/μL)		
	Normal	Suspect	Lymphocytic
0–1	<10,000	10,000–12,000	>12,000
1–2	<9,000	9,000–11,000	>11,000
2–3	<7,500	7,500–9,500	>9,500
3–4	<6,500	6,500–8,500	>8,500
>4	<5,000	5,000–7,000	>7,000

### 2.6 Data collection

For each study cow, the following data were collected: study herd identification (A–K), cow identification, age (years), sampling date(s), ELISA test result (negative, positive), WBC, and BLV status (positive, negative).

### 2.7 Data analysis and processing

To surmise transmission parameters, we compiled data using information from each farm’s records (covering a 1-year period), then estimated the most likely date of infection for new cases.

For cows that tested negative in the first sample, time-at-risk was calculated from initial sampling (left censoring) through to the estimated time of infection (as detailed below), or right-censored with the study’s end.

#### 2.7.1 Estimation of most likely time of infection and the effective transmission rate

Because the exact time each new infection occurred was unknown, we estimated the possible time of infection following the method outlined above (18). However, it is important to note that the date of the observed seroconversion does not correspond with the time of infection because cows were not bled and tested for virus detection daily. Furthermore, there is a time delay between the date of infection and when a test can detect a certain level of antibodies. Therefore, the most likely time of infection can be probabilistically deduced using the last observation of a negative serological result (*t*_k1-1_) and the time of the first positive observation (*t*_k1_) (18). Briefly, to analyze the data and obtain the most likely time of infection, we used a Bayesian approach based on the concept of census intervals for survival analysis (23). From each animal that seroconverted, the median (in days) of the probability of becoming infected was computed as a posterior distribution—with figures obtained from the prior distribution and the observed seroconversion date from the dataset. The prior gamma distribution was obtained using results from a previous study that estimated time-to-seroconversion following experimental BLV infection (2); while the dataset included the interval between samplings and the ELISA results of each cow per sampling. The median derived from the posterior distribution represented the most likely time of infection. Calculations were performed using WinBUGS V.1.4 (24).

The effective transmission rate (β) represents the number of new infections (per unit of time) generated by an infectious individual after contact with susceptible individuals (25). There are many approaches for estimating this transmission rate. One of those used more extensively involves deriving the rate from observational or experimental transmission data. The Susceptible (S)-Infectious (I) epidemic model can be used to assess in-herd infection dynamics, with a cow as a unit of interest and a transmission term βSI/N. As per the study, cows were observed in groups in a pasture-based production system, from which we assume homogeneous mixing. In this model, as explained in Velthuis et al. (17), the number of newly infected cows $E\;\left(\frac{C}{S}\right)$ in a time interval (*dt*) is approximately binomially distributed with a probability,


$$p_{\text{inf}}\left(t,\;\Delta t\right)=1-e^{-\beta_{t}*\frac{I_{t}}{N_{t}}*dt}$$

Based on this calculation, the maximum likelihood value for the transmission rate parameter β can be obtained by fitting a Generalized Linear Model (GLM) with a complementary log–log link function and log *(I(t)/N(t))* as an offset variable (17, 25).

However, the above model can be adjusted to account for heterogeneity in the infectiousness of infected individuals, as demonstrated by other authors (17). For our model, we combined the serological result and lymphocyte counts to identify two types of infectious (I) animals (high = PL and low = AL) and non-infected animals. Thus, the rate at which S are infected is given by βSI/N, and the probability of a single susceptible animal becoming infected during a time period (*dt*) following exposure to two types of infectious individuals is:


$$\mathrm{cloglog}\;E\;\left(\frac{C}{S}\right)=\alpha +\log\beta_{\mathrm{PL}}*V_{1}+\log\;\left(\frac{I_{t}}{N_{t}}\right)*dt$$

The regression coefficients can be interpreted as follows: α is the intercept and the log of the transmission rate of the reference that is direct transmission from AL individuals, and V1 is the effect of direct transmission from PL individuals.

We ran two models: a general one to obtain a total transmission rate parameter for infectious animals without discriminating their PL status, and another including both types of infected animals related to their infectiousness. The 95% confidence interval (CI) of the estimated β parameters was calculated using the standard error of the mean of log β. We performed all analyses in R software (26), using the lme4 package (27), and we evaluated the goodness-of-the-fit of the models by the Akaike Information Criteria (AIC).

#### 2.7.2 Reproduction ratio (*R*_0_)

The basic *R*_0_ is a key parameter in transmission theory, representing a threshold condition that determines whether an infectious disease will spread in a susceptible population once the disease has been introduced. In our epidemiological context, the *R*_0_ is calculated using the product of the mean infectious period (1/γ) and the effective transmission rate β (obtained from the GLM model):


R0=β∗1γ

We estimated 1/γ as the difference between the median age at infection and the life expectancy for BLV-infected cattle, because animals remain infected for the rest of their lives [based on Monti et al. (28)]. The life expectancy of AL-infected animals averaged 3.4 years, and—as it was not possible to obtain individual information from the animals—we assumed the life expectancy of PL animals to be half of those with AL, given the time taken to develop the PL condition (29).

## 3 Results

Based on the ELISA test results, 963 cows tested negative, 459 cows were infected [46.4%; 95% CI (43.3; 49.6%)], and 105 cows (24.1%) were new cases of infected cows during the follow-up period. We also identified 45 cows [10.3%; 95% CI (7.8; 13.5%)] as infected and PL (Table 2).

**Table 2:** Descriptive results of BLV-infection in cattle on 11 studied dairy farms in Chile.

Farm	Herd size (*n*)	Negative (*n*)	Infected (*n*)	New cases (*n*)	PL			Age (median) (years)^*^
					*n*	%	95% CI	
A	29	24	4	1	0	0	0	3.2
B	63	54	8	1	0	0	0	6.1
C	29	23	5	1	0	0	0	6.7
D	30	19	10	1	2	18.2	5.1; 47.7	4.4
E	219	114	92	13	7	6.7	3.3; 13.1	5.5
F	151	46	89	16	20	19.0	12.3; 27.6	5.0
G	370	252	92	26	1	0.8	0.1; 4.6	7.0
H	233	225	7	1	0	0	0	5.2
I	292	113	139	40	12	6.7	3.9; 11.3	5.5
J	82	65	15	2	3	17.6	6.2; 41.1	6.1
K	29	28	1	0	0	0	0	5.3
Total	1,527	963	459	105	45	8.0	6.1; 10.5	

^*^For herds without PL represents the median age of the herd and for those herds with PL is the median age of PL animals only.

The overall proportion of herds infected with bovine leukosis virus in dairy herds in southern Chile was 34.7% [95% CI (22.6, 44.1)] and the apparent within-herd prevalence was 14.6% (19). In the study population we found a prevalence of 30.5% (95% CI = 27.8; 32.4%), and the cumulative incidence was 10.9% (95% CI = 9.1; 13.0%), however, great variability was observed between farms for both indicators.

The overall proportion of cows that presented with PL was 8.0% (95%CI = 6.1; 10.5%) (Table 2). However, a large variability was observed between farms. Farm F presented the largest number of PL cows (*n* = 20), although the percentage of PL was similar to that observed in Farms D and J. Cows on the assessed farms that had PL were older than 3 years, which is consistent with results reported in the literature (30).

### 3.1 Effective transmission rate (β) and basic reproduction ratio (*R*_0_)

The estimated β (coefficient) was higher in PL cattle (1.1; 95% CI = −1.6, 3.8), compared to AL cattle (−3.1; 95% CI = −3.7, −2.5). In addition, the *R*_0_ was higher in PL cattle (60.4: 95% CI = 3.5; 820.6), compared to AL cattle (1.5; 95% CI = 0.7, 3.1) (Table 3).

**Table 3:** Estimated β and *R*_0_ for transmission of BLV from infected cows, PL infected cows, and AL infected cows, based on their infection status and a 95% CI.

Condition	β	95% CI	#Expected cases	95% CI	*R*_o_	95% CI
All categories grouped^*^						
From infected	−2.92	−3.23; −2.63	0.05	0.04; 0.07	1.94	1.55; 2.71
*By infectious category^#^*						
From AL	−3.13	−3.74; −2.56	0.04	0.02; 0.08	1.55	0.78; 3.10
From PL	1.17	−1.64; 3.80	3.23	0.19; 43.88	60.4	3.55; 820.56

^*^AIC = 102.4; ^#^AIC = 103.7.

## 4 Discussion

Importantly, this study demonstrates the significant difference in the risk of BLV infection transmission in PL cattle compared to those with AL. PL is a condition that can be easily monitored and collected information used to establish a risk-based BLV control program.

Greater infectivity in PL cows is reflected in the higher effective transmission rate, number of expected cases, and R_0,_ than in AL cows. These results are associated with a higher number of circulating B lymphocytes (31) and a larger proviral load compared to those with normal counts and low proviral load (8, 12, 14, 32–34)—even when the animals were exposed to the same management practices.

In this study, the *R*_0_ was higher in PL cattle (60.4) than AL cattle, which suggests that PL status is a relevant factor in the population transmission process. On average, a PL cow will infect 60 other animals with BLV, while an AL cow will only infect two. The absolute values of the estimated β and basic *R*_0_ in the study were lower than reported in previous research (18), but this might be a consequence of lower BLV prevalence in Chilean herds compared to those previously studied in Argentina. In addition, about 10% of infected animals in the present study developed a persistent lymphocytosis condition across a range of ages exceeding 3 years, which was lower than described in previous literature (30% of infected animals that are older than 3 years) (8, 35).

The findings of this study have practical relevance because they support using a simpler test to make decisions within BLV control programs, especially for herds with a large prevalence of infection and farmers or practitioners with less access to better-equipped labs. Awareness of this tool could encourage greater implementation of a test and removal control program that is more sustainable and accessible for dairy farmers in many countries. Our findings show this testing approach is successful in reducing the burden of the infection, and provides alternative criterion for selective culling, for example, when no laboratory is available for proviral load estimations.

However, the study had some limitations. First, identifying high/low infectious animals by testing viral load is more precise than the use of WBC. Second, the limited length of the study observation period (6 months) did not allow for a more extensive evaluation of PL’s impacts on the lifespan of infected animals. Further, the small sample size affected the standard error and the width of the CIs for the transmission rates.

Finally, our findings suggest that AL is still infectious, as the infectivity and the basic *R*_0_ is above 1. Therefore, a control program based only on eliminating PL would be insufficient to control or eradicate all BLV transmission in a herd. The removal of highly infectious animals with PL can be primarily used to reduce prevalence to a certain level, after which other effective approaches can be utilized, such as test and removal.

## 5 Conclusion

The estimated β and the *R*_0_ were higher in PL cattle than AL cattle. The WBC test is a viable option that can be considered for risk identification, risk assessment, and risk management of BLV infection in dairy herds; particularly in livestock regions where laboratory capacity is limited (e.g., use of PCR or gene sequencing techniques).

## Data availability statement

The raw data supporting the conclusions of this article will be made available by the authors, without undue reservation.

## Ethics statement

The animal studies were approved by Comité de Bioética “Uso de animales en la investigación” Universidad Austral de Chile. The studies were conducted in accordance with the local legislation and institutional requirements. Written informed consent was not obtained from the owners for the participation of their animals in this study because it was not needed at the time of the study.

## Author contributions

BB: Data curation, Formal analysis, Investigation, Validation, Visualization, Writing – original draft, Writing – review & editing. GM: Conceptualization, Formal analysis, Funding acquisition, Methodology, Supervision, Writing – original draft, Writing – review & editing.
